# Clinical Outcomes of Endoscopic Resection for Large (>3 cm) Ampullary Tumors

**DOI:** 10.1055/a-2961-1504

**Published:** 2026-09-23

**Authors:** Tian-Yu Zhang, Zhang-Han Chen, Dong-Li He, Jia Song, Lin-Da Li, Han-Xiao Zhang, Yun-Shi Zhong, Yan Wang

**Affiliations:** 1 Endoscopy center 92323 Zhong Shan HospitalShanghaiShanghaiChina; 2 Endoscopy center 117880 Shanghai Xuhui Central HospitalShanghaiShanghaiChina; 3 Endoscopy center 92323 Zhongshan Hospital Fudan UniversityShanghaiShanghaiChina; 4 Department of GastroenterologyRongcheng Hospital of Traditional Chinese MedicineWeihaiShandongChina

**Keywords:** endoscopy upper GI tract, precancerous conditions & cancerous lesions (dysplasia and cancer) stomach, endoscopic resection (ESD, EMRc, …)

## Abstract

**Background and Study Aims** Current guidelines recommend en bloc endoscopic resection (ER) for ampullary tumors up to 3 cm and surgery for those exceeding 4 cm, based on low-quality evidence. Robust clinical data and operative experience with ER for tumors >3 cm are relatively limited. This study aimed to investigate the safety and efficacy of ER for large ampullary tumors exceeding 3 cm.

**Patients and Methods** This retrospective study included 28 patients with large ampullary tumors (>3 cm) treated with ER at two tertiary academic endoscopy centers from September 2017 to July 2025. Clinical, endoscopic, and pathological records and follow-up data were collected and analyzed.

**Results** All 28 patients successfully underwent ER with a complete ER rate of 100% and an R0 resection rate of 67.9%. The overall postoperative delayed bleeding rate was 14.3% (4/28; 95% CI: 5.7–31.5%). In subgroup data, bleeding occurred in 2 of 24 patients (8.3%; 95% CI: 1.5–25.8%) with stent placement, in 2 of 4 patients (50.0%; 95% CI: 8.9–91.1%) without stent placement; bleeding occurred in 3 of 27 patients (11.1%; 95% CI: 3.9–28.1%) with wound closure, and in the single patient without wound closure (100%; 95% CI: 5.1–100.0%). All bleeding events were managed endoscopically. No other adverse events occurred. After a mean follow-up of 27.4 months (range 10.0–97.0 months), two patients (7.1%) developed recurrence and underwent surgical resection ( *n* = 1) or repeat ER ( *n* = 1).

**Conclusions** ER appears safe and effective for ampullary tumors exceeding 3 cm. The potential roles of wound closure and stent placement in reducing adverse events require validation in larger comparative trials.

## Introduction

The widespread application of gastroscopy has led to increased detection of ampullary tumors, particularly in East Asian populations. 1 2 Adenomas exceeding 2 cm, whether tubular or villous, carry a significant risk of malignancy and thus warrant timely resection. 3 4 While surgical interventions, such as transduodenal ampullectomy or pancreaticoduodenectomy, were historically considered the standard treatment, their high invasiveness and prolonged recovery times have increasingly prompted a shift toward endoscopic resection (ER). 5 6 7 8 9

Current guideline from the European Society of Gastrointestinal Endoscopy recommends endoscopic en bloc resection for ampullary tumors <3 cm, primarily using snare papillectomy. For lesions >4 cm, surgery is generally advised (weak recommendation, low-quality evidence) due to the heightened technical challenges of en bloc resection; for those 3–4 cm, decisions are made on an individualized basis. 10 Although several recent studies have reported clinical outcomes including large ampullary tumors (>3 cm), there remains a lack of attention to the specific operative techniques (e.g., precutting procedures) and comprehensive perioperative management for these complex lesions. 11 12 To date, robust clinical data and operative experience for ER in tumors >3 cm remain relatively limited.

Given the minimal invasiveness of ER relative to surgery, 9 assessing its safety and efficacy for large ampullary tumors is of paramount clinical importance. This study retrospectively analyzed clinical data from patients who underwent ER for large ampullary tumors exceeding 3 cm, using precutting endoscopic mucosal resection (EMR) or precutting endoscopic piecemeal mucosal resection (EPMR), combined with pancreaticobiliary stenting and prophylactic wound closure. We aim to evaluate the safety and efficacy of this strategy, with a focus on adverse events and long-term recurrence, and to provide our practical insights for similar endoscopic procedures.

## Methods

### Study Design and Patients

This retrospective dual-center observational study was to investigate the safety and efficacy of ER for large ampullary tumor patients at two tertiary academic endoscopy centers. This investigation was approved by the Committee of Medical Ethics at Zhongshan Hospital Fudan University (number B2020-265R).

From September 2017 to July 2025, consecutive patients who underwent ER of large ampullary tumors at our centers were included in the study. Indications for ER were as follows: (1) a lesion with a maximum diameter exceeding 3 cm; (2) no clinical or imaging evidence of biliary or pancreatic duct invasion, lymph node metastasis, or distant metastasis; (3) an intact mucosal surface on endoscopic examination, without signs of malignancy such as ulceration or friability; (4) preoperative biopsy pathology showing no more than high-grade intraepithelial neoplasia (HGIN); and (5) the patient provides informed consent for ER. Cases meeting the above criteria may be considered suitable for ER aiming for complete macroscopic lesion removal. As ER of large ampullary tumors is technically complex, the procedure should be performed by endoscopists with substantial expertise in ampullary tumor ER.

A total of 28 patients were enrolled and followed up until April 2026.

### Preoperative Examination

All patients underwent comprehensive preoperative evaluation to assess tumor extent and exclude advanced disease. Duodenoscopy was performed to evaluate the macroscopic morphology and obtain biopsy specimens. Endoscopic ultrasonography (EUS) was used as the primary imaging modality to assess the depth of tumor infiltration (T stage), regional lymph node status (N stage), and the presence of biliary or pancreatic ductal extension and dilation. Contrast-enhanced computed tomography (CT) of the chest, abdomen, and pelvis was performed to rule out distant metastasis (M stage). Magnetic resonance cholangiopancreatography and intraductal ultrasonography were not routinely used at our center.

The criteria for excluding biliary or pancreatic ductal extension were based on EUS findings: (1) no continuous hypoechoic mass extending into the common bile duct or main pancreatic duct; and (2) intact ductal wall layers without disruption or irregular thickening.

The criteria for excluding invasive disease were: (1) preoperative biopsy pathology showing no more than HGIN, without definitive features of invasive adenocarcinoma (e.g., desmoplastic reaction or irregular fused glands); and (2) EUS showing tumor limited to the mucosa/submucosa (no infiltration beyond the muscularis propria) and no suspicious regional lymph node involvement.

### Endoscopic Procedures

After standard preoperative preparation, an Olympus electronic endoscopy system with a TJF-260V electronic duodenoscopy (Olympus Corp., Japan) was used for ER and the subsequent endoscopic retrograde cholangiopancreatography. Primary instruments included a KD-620LR electrosurgical knife, a KD-611L IT knife and HX series snares (Olympus Corp., Japan), along with an Erbe VIO 200D high-frequency electrosurgical unit and a VIO APC2 argon plasma coagulation system (ERBE Elektromedizin GmbH, Germany). Procedures were performed under general anesthesia. If necessary, hyoscine was administered intraoperatively to suppress intestinal peristalsis.

ER technique included the following approaches: (1) Precutting EMR ( Fig. 1 ): Submucosal injection (4% indigo carmine with 1:100,000 epinephrine in normal saline) was performed to lift the anal or oral side of the tumor, as well as the extrapapillary component of the large ampullary tumor when present. A partial mucosal incision was then made using a hook knife at the previously injected site on either the anal or oral side of the lesion, which was followed by en bloc snare resection, with the snare leveraging the incised margin as a fulcrum. (2) Precutting EPMR ( Fig. 2 ): Submucosal injection and partial mucosal incision were performed as described above. The lesion was then resected piecemeal using a snare. Of note, the choice between precutting EMR and precutting EPMR was determined by whether the lesion could be completely ensnared after the full release of a snare.

**Fig. 1:**
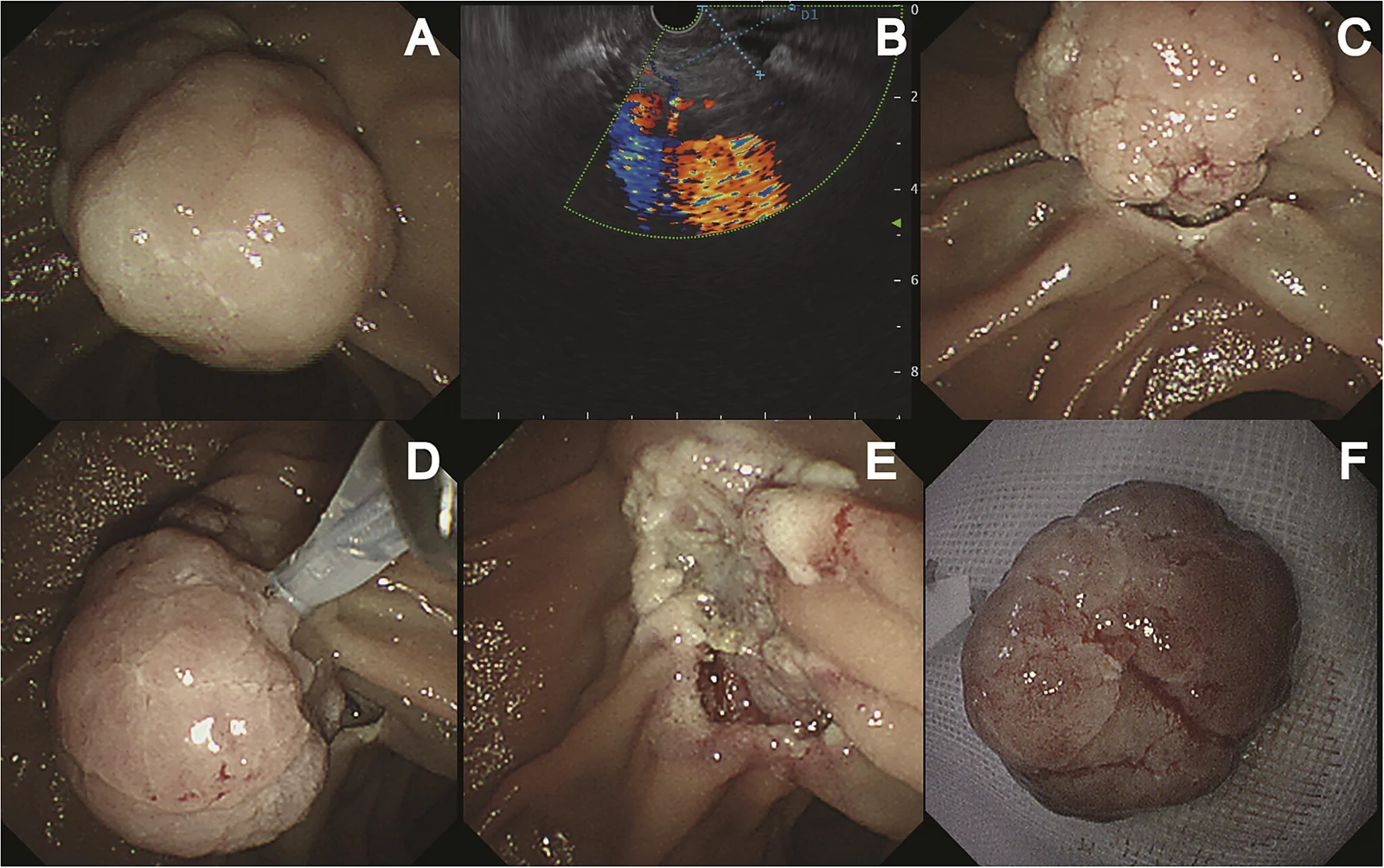
Precutting EMR for large ampullary tumor. ( **A** ) Lesion; ( **B** ) Preoperative EUS of the lesion; ( **C** ) Submucosal injection on the anal side of the lesion and partial mucosal incision; ( **D** ) Snare resection; ( **E** ) Postoperative wound; ( **F** ) Specimen.

**Fig. 2:**
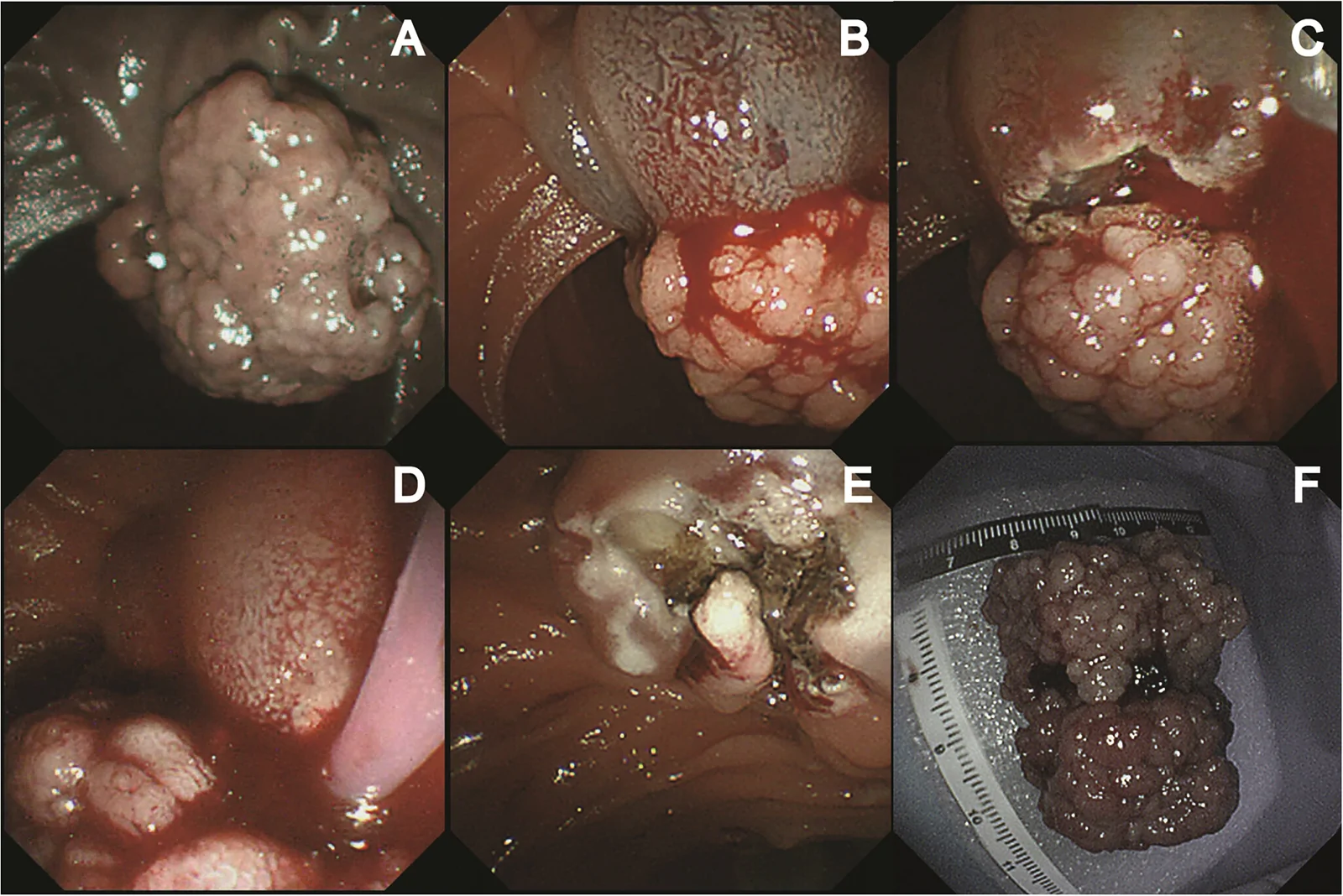
Precutting EPMR for large ampullary tumor. ( **A** ) Lesion; ( **B** ) Submucosal injection on the oral side of the lesion; ( **C** ) Partial mucosal incision; ( **D** ) Snare resection; ( **E** ) Postoperative wound; ( **D** ) Specimens.

Technical details of submucosal injection on the anal or oral side of the lesion: The choice of submucosal injection and precutting site was dictated by the endoscopic visibility of the anal (lower) margin under side-viewing. When the anal margin was clearly exposed ( Fig. 1c ), injection and precutting were technically feasible on this side, creating a reliable fulcrum that stabilized the snare and prevented inadvertent capture of the normal anal-side duodenal mucosa. This approach was generally adopted for lesions scheduled for en bloc resection using the precutting EMR technique. Conversely, when the tumor was so large that the anal margin was obscured or anatomically inaccessible for injection ( Fig. 2a ), the oral (upper) margin was selected as an alternative anchoring point for the snare. This strategy was often employed for piecemeal resection, proceeding in an oral-to-anal direction.

The principles of managing the presence of limited extrapapillary adenomatous extension: When the en bloc resection was not achievable, the papillary component was resected first en bloc or piecemeal after submucosal injection and mucosal precutting, and the extrapapillary extension was then addressed subsequently. This sequential strategy ensures complete and safe resection of the ampullary component of the tumor, as well as the protection of the pancreaticobiliary orifice.

Biliary and pancreatic duct stents (Boston Scientific Corp., USA) were placed as needed. Placement of stents (biliary, pancreatic, or both) was performed only after successful guidewire cannulation of the respective duct, with a maximum of three attempts per duct. Wound closure was routinely performed with metal clips and partial closure was performed in some cases ( Fig. 3 ).

**Fig. 3:**
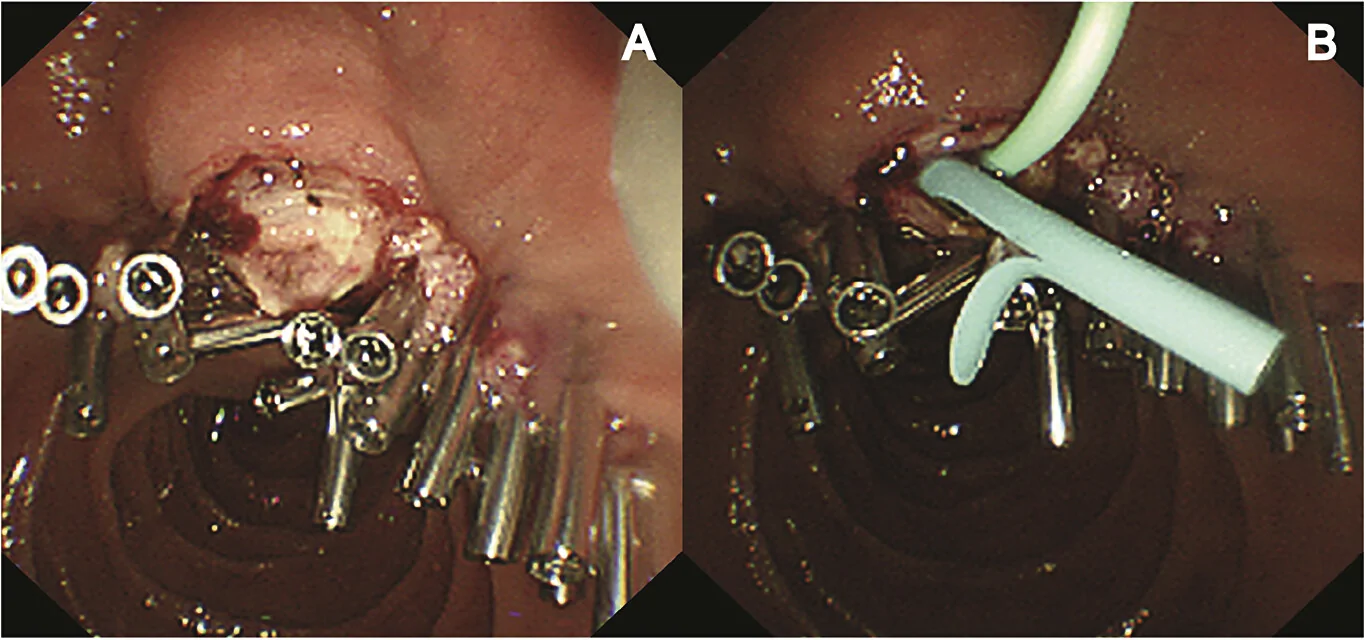
Wound closure and stent placement after ER of large ampullary tumor. ( **A** ) Wound closure with metal clips; ( **B** ) Placement of the biliary and pancreatic stents.

Postoperative management: A nasoduodenal (ND) tube was placed for effective gastrointestinal decompression and timely detection of wound delayed bleeding. During the strict fasting period, patients received intravenous antibiotics, proton pump inhibitors and somatostatin. Analgesics and antiemetics were administered as needed for postoperative pain and vomiting. Laboratory monitoring included complete blood count, liver and renal function tests, and serum amylase/lipase levels on postoperative days 1 and 3, supplemented by imaging evaluations as clinically indicated.

### Pathology

For postoperative pathological evaluation, routine cases were reported by attending or senior pathologists (associate chief physician or above). Complex or challenging cases were jointly reviewed by at least two senior pathologists and consensus was required before the final report was issued. For malignant tumor diagnoses, a “double-review and double-signature” protocol was followed, particularly for diagnoses that affect surgical planning or adjuvant therapy decisions. Intraoperative frozen section analysis was not routinely performed at our centers.

For patients whose postoperative pathology revealed intramucosal carcinoma, the patients and their families were informed of the findings and additional surgical intervention (transduodenal ampullectomy) was recommended.

### Outcomes and Definitions

Primary outcomes were the en bloc resection rate (technical success defined as removal of the entire target lesion in one piece without fragmentation) and the complete ER rate (technical success defined as the absence of any adenomatous remnant from the resection margins at the end of the procedure, as judged by the operating endoscopist). 10 Secondary outcomes included the incidence and severity of adverse events (graded as severe and mild, according to the necessity of repeat endoscopic intervention), 13 the recurrence rate during follow-up and procedure time (defined as the total endoscopic operative time from the start of submucosal injection to the final placement of the ND tube, including tumor resection, clip closure, possible pancreaticobiliary cannulation, stents placement, and ND tube placement).

Adverse events were defined as follows: (1) delayed bleeding: bleeding from the wound occurring more than 24 h after the procedure, manifested as bloody drainage from the ND tube, hematemesis, melena, or even hypovolemic shock; (2) postoperative pancreatitis: new or worsening abdominal pain persisting for more than 24 h postoperatively, with serum amylase or lipase at least three times the upper limit of normal, and resulting in prolonged hospital stay; and (3) postoperative perforation: sudden onset of severe abdominal pain and peritoneal signs, with imaging (CT or X-ray) demonstrating free gas in the retroperitoneal or peritoneal cavity.

### Follow-up

For patients undergoing ER of ampullary tumors, standardized endoscopic surveillance, including biopsies of the scar and any abnormal area, was performed postoperatively at 3, 6, and 12 months, and then annually for at least 5 years. For patients with histopathologically confirmed early cancer, systematic follow-up should be performed. It is mandatory to perform at least four duodenoscopies combined with imaging evaluations within the first 24 months postoperatively, with subsequent monitoring frequency adjusted according to the patient’s recovery status. EUS was not performed routinely but was selectively indicated when: (1) visible recurrence was suspected at surveillance endoscopy, or (2) histopathology of scar biopsies revealed low-grade intraepithelial neoplasia (LGIN) or higher grade dysplasia.

Follow-up duration was calculated from the date of ER to the last clinical contact or the study cutoff date (April 2026).

### Data Collection

Data on patient demographics (age, sex), tumor diameter, and procedural details (resection technique, operative duration, stent placement and wound closure) were retrieved from electronic medical records. Postoperative pathology included lesion type and grade of intraepithelial neoplasia. All ER-related adverse events were recorded.

### Statistical Analyses

Statistical analysis was performed using SPSS 21.0 (IBM Corp., USA). Quantitative variables were expressed as means and standard deviations (SDs), and categorical parameters were expressed as numbers, frequencies and 95% confidence intervals (CIs). Owing to the retrospective, nonrandomized design and the markedly imbalanced subgroup sizes, no formal statistical comparisons (e.g., chi-square or Fisher’s exact tests) were performed between subgroups; all results are presented as descriptive.

## Results

### Clinicopathologic and Demographic Features of the Patients

The clinical features of all patients are shown in Table 1 . Briefly, the 28 patients (mean age, 59.7 ± 11.9 years, 57.1% male) were treated with ER from September 2017 to July 2025, and the mean lesion diameter was 4.8 ± 1.4 (median: 4.5, range: 3.5–10.0) cm.

**Table 1:** Demographic and clinicopathological data of 28 patients, *n* (%).

Parameter	Total ( *n* = 28)	Precutting EMR ( *n* = 12)	Precutting EPMR ( *n* = 16)
Sex			
Female	12 (42.9)	5 (41.7)	7 (43.8)
Male	16 (57.1)	7 (58.3)	9 (56.3)
Age, mean ± SD (year)	59.7 ± 11.9	57.8 ± 14.1	61.1 ± 10.1
Lesion size (cm)	4.8 ± 1.4	4.3 ± 0.8	5.2 ± 1.6
Operation duration, mean ± SD (min)	53.0 ± 26.0	52.5 ± 28.9	53.8 ± 25.1
Postoperative pathological findings			
Tubular adenoma with LGIN	6 (21.4)	2 (16.7)	4 (25)
Tubular villous adenoma with LGIN	7 (25.0)	3 (25)	4 (25)
Conventional serrated adenoma with LGIN	1 (3.6)	1 (8.3)	0
Villous adenoma with HGIN	1 (3.6)	1 (8.3)	0
Tubular adenoma with HGIN	2 (7.1)	1 (8.3)	1 (6.3)
Tubular villous adenoma with HGIN	8 (28.6)	2 (16.7)	6 (37.5)
Tubular adenocarcinoma	1 (3.6)	0	1 (6.3)
Villous adenocarcinoma	2 (7.1)	2 (7.1)	0
Margin status			
Horizontal margin			
Negative	23 (82.1)	12 (100)	11 (68.8)
Positive	0	0	0
Indeterminate/unevaluable	5 (17.9)	0	5 (31.3)
Vertical margin			
Negative	19 (67.9)	10 (83.3)	9 (56.3)
Positive	2 (7.1)	2 (16.7)	0
Indeterminate/unevaluable	7 (25)	0	7 (43.8)
R0 resection	19 (67.9)	10 (83.3)	9 (56.3)
Complete endoscopic resection	28 (100.0)	12 (100)	16 (100)
En bloc resection	12 (42.9)	12 (100)	0
Recurrence	2 (7.1)	1 (8.3)	1 (6.3)

EMR: endoscopic mucosal resection; EPMR: endoscopic piecemeal mucosal resection; LGIN: low-grade intraepithelial neoplasia; HGIN: high-grade intraepithelial neoplasia.

Among the 28 patients, 12 underwent precutting EMR, 16 underwent precutting EPMR. The en bloc resection rate was 42.9% (12/28), and the complete ER rate was 100.0% (28/28). In one case, the mucosal defect was not clipped, and no pancreatic or biliary stent was placed. In the remaining 27 cases, the defect was partially or completely closed with metal clips. Among these, 24 cases received stent placement: 11 with biliary stents only, 3 with pancreatic stents only, and 10 with both biliary and pancreatic stents.

Regarding postoperative recovery, ND tubes were placed in all 28 patients (100%) for early enteral nutrition and monitoring of delayed bleeding, with a mean indwelling time of 12.8 ± 4.6 (range: 1–22) days. The mean postoperative hospital stay was 16.5 ± 7.8 (range: 4–23) days. No patient required readmission within 30 days postdischarge.

Postoperative pathological examination of the 28 lesions revealed the following histological types: 8 tubular adenomas, 1 villous adenoma, 15 tubulovillous adenomas, 1 traditional serrated adenoma, 1 tubular adenocarcinoma, and 2 villous adenocarcinomas. Among the 25 adenomas (noncancerous lesions), 23 showed LGIN, including nine with focal HGIN; the remaining two adenomas exhibited HGIN. The three adenocarcinomas were all well-differentiated intramucosal cancers (pTis). In two of them, HGIN components were present adjacent to the resection margin without vertical margin positivity, deep submucosal invasion or lymphovascular and perineural invasion; both patients declined additional surgery or chemoradiotherapy.

### Adverse Events

A total of four adverse events (14.3%, 95% CI: 5.7–31.5%) occurred among the 28 patients, all delayed bleeding. The description of postoperative delayed bleeding according to the wound closure and stent placement is shown in Table 2 . No perforation, pancreatitis, cholangitis, or papillary stenosis was observed. When grouped by wound closure, delayed bleeding occurred in 3 of 27 patients (11.1%; 95% CI: 3.9–28.1%) with clipped wounds, and in the single patient without clipping (100%; 95% CI: 5.1–100.0%). When grouped by stent placement, bleeding occurred in 2 of 24 patients (8.3%; 95% CI: 1.5–25.8%) with stents, versus two of four patients (50.0%; 95% CI: 8.9–91.1%) without stents.

**Table 2:** Postoperative delayed bleeding rates by wound closure and stent placement.

Group	*n*	Delayed bleeding, *n* (%)	95% CI
Wound closure			
Clipped	27	3 (11.1)	3.9–28.1
Unclipped	1	1 (100)	5.1–100
Stent placement			
With stent	24	2 (8.3)	1.5–25.8
Without stent	4	2 (50)	8.9–91.1
Combined subgroups			
Unclipped wound + no stent	1	1 (100)	5.1–100
Clipped wound + no stent	3	1 (33.3)	1.7–88.2
Clipped wound + biliary stent	11	0	0–25.9
Clipped wound + pancreatic stent	3	0	0–56.1
Clipped wound + pancreatic and biliary stents	10	2 (20)	3.5–51.0
Total	28	4 (14.3)	5.7–31.5

CI: confidence interval.

### Follow-up

All patients were followed up until April 2026, with a mean follow-up duration of 27.4 months (range: 10.0–97.0 months). Two patients experienced recurrence during follow-up: one with initial carcinoma in situ and HGIN component abutting the margin (recurrence at 18 months, treated surgically), and the other with tubular adenoma and LGIN with negative margins (recurrence at 24 months, treated endoscopically). No patients developed lymph node or distant metastasis. No postoperative deaths occurred.

## Discussion

With the continuous advancement of ER techniques, ER for ampullary tumors has been increasingly widespread. However, due to limited relevant studies and variations in experience among different centers, there is still no consensus on the maximum effective diameter for ER of ampullary lesions. Traditionally, large tumors have been more commonly recommended for surgical resection. 10 Therefore, we provide our endoscopic experience and clinical outcomes here.

All 28 ERs for ampullary tumors were successfully completed, with a success rate comparable to ER for smaller lesions. Among them, complete ER was achieved in 28 cases (100%), and en bloc resection was achieved in 12 cases (42.9%). The former one is comparable to the outcomes reported in previous studies on small-sized lesions (100% vs. 94.2%), while the later one is lower than currently reported outcomes (42.9% vs. 82.4%) due to the application of EPMR for large lesions. 14 Two patients experienced recurrence after the procedure, yielding a recurrence rate of 7.1% (2/28), which is also comparable to the recurrence rates reported in studies on ER of lesions smaller than 3 cm. 15 16 Additionally, the average procedure time for large lesions was 53.0 min, compared to 36.7 min for smaller lesions during the same period at our center. This difference may be attributed to the larger wound surface left after resection of large lesions, which increases the difficulty of closure, the number of clips required, and thus the overall procedure time.

Although ER offers the advantage of being minimally invasive, adverse events may still occur due to the complex anatomy of the procedure site. 12 Common early postoperative adverse events include bleeding, perforation, and acute pancreatitis, while late adverse events mainly involve localized strictures at the biliary or pancreatic duct orifices. 13 Among these, bleeding and acute pancreatitis are the most frequent adverse events following ER of ampullary tumors, with reported incidence rates of 10.6% and 11.9%, respectively, in previous studies involving small lesions. 14 In our study, the postoperative bleeding rate was 4 out of 28 cases (14.3%). Although this is slightly higher than that observed with resections of smaller lesions, there were no instances of perforation, acute pancreatitis, or localized strictures of the biliary or pancreatic duct orifices. Moreover, a pooled analysis involving 4935 patients reported an overall adverse event rate of 30.0% following resection of small lesions. 17 In our study, the overall adverse event rate was 14.3%, which is quite lower than the previously reported result. Compared with the recently reported ESDIP technique, 18 the precutting EMR/EPMR strategy in our study appears to offer a simplified procedural workflow and a potential intraoperative safety advantage, with a 0% perforation rate versus 15% reported for ESDIP. This difference is likely attributable to the lower mechanical tension exerted on the duodenal wall by snare resection. Regarding postoperative complications, our delayed bleeding rate (14.3%) was numerically comparable to that of ESDIP (19%). However, we acknowledge that piecemeal specimens from EPMR are prone to higher rates of positive or unevaluable margins, which may compromise pathological assessment and adjuvant decision-making—a clear drawback compared with en bloc ESDIP specimens. Nonetheless, rigorous preoperative examination, regular surveillance, and timely repeated resection may partly offset this limitation. In terms of clinical applicability, precutting EMR/EPMR may be more suitable for protruded-type large ampullary adenomas, whereas ESDIP may offer advantages for flat-type mucosal lesions involving the papilla with circumferential spread along the duodenal wall, suggesting a complementary, morphology-tailored strategy in future practice. Finally, more evidence is warranted for the comparison and promotion of these two different techniques.

In this study, the incidence of postoperative bleeding was 14.3% (4/28, 95% CI: 5.7–31.5%). Numerically, the bleeding rate appeared lower in patients who underwent wound closure (11.1%, 3/27) compared with the single patient who did not (100%, 1/1), and also appeared lower in those with stent placement (8.3%, 2/24) than in those without (50.0%, 2/4). However, given the retrospective, nonrandomized design and the extremely small subgroup sizes, reliable statistical comparisons between groups were not feasible, and the current evidence is insufficient to establish a protective effect of wound closure or stent placement against postoperative delayed bleeding. Some studies have suggested that the placement of a pancreatic stent did not appear to have significant impact on postoperative bleeding, which may be related to the presence of an accessory pancreatic duct, 19 and ER generally does not impair pancreatic juice drainage or lead to erosion of the resection surface. 20 Nevertheless, these hypotheses remain unsubstantiated by our data. Therefore, the clinical value of routine wound closure and stent placement in preventing bleeding after ampullary tumor resection remains uncertain, and further well-designed prospective randomized studies are warranted. Furthermore, repeated attempts to place a pancreatic stent may increase the risk of postoperative pancreatitis 21 ; therefore, at our center, pancreatic stents are not routinely placed in patients whose resection wounds are closed with clips. Notably, a previous study conducted at our center showed that wound closure could completely prevent postoperative bleeding in ampullary lesions ≤3 cm, 22 while in large lesions, it appears to be less effective in preventing bleeding, possibly due to the larger surface area of the resection wound. This suggests that the closure technique may need to be refined based on the wound size. However, given the limited sample sizes in both studies, the conclusion is not definitive and requires further investigation and validation. In addition, our center has summarized a vascular strategy that may help reduce the incidence of postoperative bleeding: Intraoperatively, targeted hemostasis is applied to the 3- to 9-o’clock position of the papilla, and a refined coagulation mode is recommended for this area, which accounts for approximately 45% of its arterial blood supply. Anatomical studies have confirmed that the 10- to 11-o'clock position is relatively avascular, which may thus serve as a safer operative window. 23 Also, we propose that submucosal injection and partial mucosal incision on the anal or oral side of the tumor can provide an incised edge that serves as a convenient fulcrum for subsequent snare resection, which can effectively reduce the difficulty of using a snare to achieve complete ER in this anatomical space.

Our center has also summarized the following key experience regarding postoperative management and early detection of adverse events: (1) duration of NG tube placement should be adjusted based on the patient’s postoperative risk level and is not necessarily associated with the occurrence of adverse events. Currently, there is no consensus on the optimal duration of NG tube placement following this type of endoscopic procedure. 10 In this study, NG tube placement lasted from 1 to 22 days, with 23 out of 28 patients (82.1%) having the tube in place for more than 10 days. The median duration was 14 days, which reflects the relatively high postoperative risk associated with ampullary tumor resection. (2) At our center, oral water intake typically resumes the day before NG tube removal, which is another important consideration in determining NG tube duration. In this study, due to the larger resection wound and slower healing process, the time to resume oral water intake was often extended to more than 8 days. (3) NG tube placement plays an important role in the early detection of delayed postoperative bleeding, 3 as it allows for the drainage of both digestive fluids and any blood-tinged fluid resulting from delayed bleeding. Therefore, prolonged NG tube placement can enhance the timely identification and management of postoperative adverse events, contributing to improved recovery outcomes. Thus, the decision regarding NG tube duration should be made in conjunction with an assessment of postoperative risk and monitoring needs.

However, due to the limited number of cases, the evidence remains insufficient. Furthermore, this study is retrospective in nature and lacks a comparative cohort of small lesions treated endoscopically during the same period at our center. Thus, further studies with larger sample size and multiarm design are warranted.

## Conclusions

In summary, ER appears safe and effective for large ampullary tumors exceeding 3 cm, achieving favorable rates of complete ER, adverse events, and long-term recurrence. These outcomes are comparable to those reported for smaller tumors before, despite the larger lesion size. Therefore, ER may be considered a feasible treatment option for large ampullary tumors ranging from 3.5 to 10 cm. However, the potential roles of wound closure and stent placement in reducing adverse events require validation in larger comparative trials.

## Declaration of GenAI Use

The authors confirm that no Generative AI was used in the preparation of this manuscript.
